# Two Beats One: Osteosarcoma Therapy with Light-Activated and Chemo-Releasing Keratin Nanoformulation in a Preclinical Mouse Model

**DOI:** 10.3390/pharmaceutics14030677

**Published:** 2022-03-19

**Authors:** Elisa Martella, Barbara Dozza, Claudia Ferroni, Clement Osuru Obeyok, Andrea Guerrini, Daniele Tedesco, Ilse Manet, Giovanna Sotgiu, Marta Columbaro, Marco Ballestri, Lucia Martini, Milena Fini, Enrico Lucarelli, Greta Varchi, Serena Duchi

**Affiliations:** 1 Institute for the Organic Synthesis and Photoreactivity (ISOF), National Research Council (CNR), 40129 Bologna, Italy; elisa.martella@isof.cnr.it (E.M.); claudia.ferroni@isof.cnr.it (C.F.); clement.osuruobeyok@gmail.com (C.O.O.); andrea.guerrini@isof.cnr.it (A.G.); daniele.tedesco@isof.cnr.it (D.T.); ilse.manet@isof.cnr.it (I.M.); giovanna.sotgiu@isof.cnr.it (G.S.); marco.ballestri@isof.cnr.it (M.B.); 2 Rizzoli Laboratory Unit, Department of Biomedical and Neuromotor Sciences (DIBINEM), Alma Mater Studiorum University of Bologna, 40123 Bologna, Italy; barbara.dozza@ior.it; 3 Electron Microscopy Platform, IRCCS Istituto Ortopedico Rizzoli, 40136 Bologna, Italy; marta.columbaro@ior.it; 4 Complex Structure Surgical Sciences and Technologies, IRCCS Istituto Ortopedico Rizzoli, 40136 Bologna, Italy; lucia.martini@ior.it (L.M.); milena.fini@ior.it (M.F.); 5 Regenerative Therapies in Oncology of the Osteoncology, Bone and Soft Tissue Sarcomas and Innovative Therapies Unit, IRCCS Istituto Ortopedico Rizzoli, 40136 Bologna, Italy; enrico.lucarelli@ior.it; 6 Department of Surgery, St. Vincent’s Hospital Melbourne, University of Melbourne, Fitzroy, VIC 3065, Australia

**Keywords:** osteosarcoma, musculoskeletal tumors, drug delivery, photodynamic therapy, keratin nanoparticles, chemotherapy, orthotopic osteosarcoma murine model

## Abstract

Osteosarcoma treatment is moving towards more effective combination therapies. Nevertheless, these approaches present distinctive challenges that can complicate the clinical translation, such as increased toxicity and multi-drug resistance. Drug co-encapsulation within a nanoparticle formulation can overcome these challenges and improve the therapeutic index. We previously synthetized keratin nanoparticles functionalized with Chlorin-e6 (Ce6) and paclitaxel (PTX) to combine photo (PDT) and chemotherapy (PTX) regimens, and the inhibition of osteosarcoma cells growth in vitro was demonstrated. In the current study, we generated an orthotopic osteosarcoma murine model for the preclinical evaluation of our combination therapy. To achieve maximum reproducibility, we systematically established key parameters, such as the number of cells to generate the tumor, the nanoparticles dose, the design of the light-delivery device, the treatment schedule, and the irradiation settings. A 60% engrafting rate was obtained using 10 million OS cells inoculated intratibial, with the tumor model recapitulating the histological hallmarks of the human counterpart. By scheduling the treatment as two cycles of injections, a 32% tumor reduction was obtained with PTX mono-therapy and a 78% reduction with the combined PTX-PDT therapy. Our findings provide the in vivo proof of concept for the subsequent clinical development of a combination therapy to fight osteosarcoma.

## 1. Introduction

Osteosarcoma (OS) is the most common primary bone tumor affecting children and young adults, with an incidence of 3–4 cases/million/year worldwide [1]. The current standard of care for OS treatment includes a combination of surgery and multi-drug chemotherapy. The purpose of the surgery is to remove the tumor with adequate surgical margins and, at the same time, preserve the best function of the operated part and prevent recurrence and metastasis [2]. The pre- and post-operative multi-agent chemotherapy, consisting of doxorubicin, methotrexate, and cisplatin, allows to induce cell necrosis, define better margins, and improve the surgical options and the outcome [3]. The introduction of neo adjuvant chemotherapy and the advance in surgical technologies increased the 5-year survival rate of patients to 60%, with no meaningful improvement achieved in the past 30 years, and this plummets to 20% if the patient develops pulmonary metastases [2]. Unfortunately, the insurgence of multi-drug resistance (MDR) limits the increase of success rate up to 100%. The most characterized mechanism of MDR is the overexpression, by tumor cells, of the ATP binding cassette transporter B1 (ABCB1), a transmembrane protein that effluxes doxorubicin and other chemotherapeutic drugs out from the cells, decreasing their intracellular accumulation and toxicity. Moreover, recent studies have demonstrated that other drug transport mechanisms, such as the reduced folate carrier, and specific signal transduction pathways, play an important role in OS chemo-resistance [4], making the management of OS patients extremely challenging [5]. Considering the limitations of standard chemotherapy, the future of OS treatment is moving towards a multimodal strategy that combines two or more therapeutic agents to enhance their efficacy in a synergistic or additive manner [6]. Nevertheless, combination therapies present distinctive challenges such as increased toxicity and uneven exposure to specific drug ratios that can lead to MDR and hamper the treatment effectiveness [7]. To this purpose, in the past decade, nanoparticles have emerged as promising carriers for the co-delivery of multiple drugs to improve drug solubility, reduce systemic toxicity and MDR, increase circulation times in the blood, control the release profiles, and target specific cells and tissues [8,9].

In a previous study, we designed and characterized nanoformulations made of high molecular weight and water-soluble keratin covalently functionalized with the photosensitizer Chlorin-e6 (Ce6) and co-loaded with paclitaxel (PTX), as a novel multimodal approach for the pharmacological treatment of OS. These bimodal nanoparticles, combining chemo- and photo-dynamic therapies, were generated with the aim to specifically deliver PTX to cancer cells, while decreasing its general toxicity and scarce intracellular accumulation. The combined therapy aims to provide a second therapeutic agent to wipe out OS cells, which might have survived the PTX or become chemo-resistant, via photodynamic therapy (PDT) [10]. Similar formulations have been designed and characterized to improve PTX solubility, reduce its systemic toxicity, and achieve a synergistic effect with the use of oxygen radicals generated by specific light-activated molecules. Park and co-authors, for instance evaluated the cytotoxic effect of PDT combined with PTX on gastric cancer cells (NCI-N87) and bile duct cancer cells (YGIC-6B) using verteporfin and a 665–675 nm irradiation light [11]. In vitro analyses showed that PTX pre-treatment enhances PDT-mediated cancer therapy due to an augmented apoptotic response mediated by cytochrome c released from mitochondria without Bax or Bid activation.

Thapa et al. generated a far-red light-activatable prodrug of PTX by conjugating a zinc phthalocyanine photosensitizer via a singlet oxygen-cleavable amino-acrylate linker [12]. In the dark, the prodrug showed a reduced cytotoxicity compared to irradiated cells. Once illuminated with far-red light it effectively killed SKOV-3 ovarian cancer cells through the combined effects of PDT and locally released PTX.

In their study, Chang et al. combined PDT and chemotherapy in one platform creating a porphyrin-lipid nano-emulsion with PTX loaded in the oil core (PLNE-PTX) [13]. Data from xenograft KB-tumor-bearing mice showed high tumor accumulation of PLNE-PTX and high inhibitory effect on tumor growth (78%) 16 days post treatment via additive effect, with monotherapy resulting in 44% and 46% tumor reduction for PDT and chemotherapy, respectively.

In the present study, we evaluate the anti-tumor effect of PTX-PDT combined therapy delivered in a bone tumor using an osteosarcoma murine preclinical model. The development of a reliable and robust preclinical OS model represents a critical factor to evaluate the efficacy of a pharmacological treatment. In the past, murine OS models were induced by exposing rodents to high doses of chemical or radioactive carcinogens [14,15]. This practice is nowadays dismissed because of the unpredictability on the tumor growth, in terms of origin sites and developmental dynamics, with the consequent inability to characterize the tumor itself [15]. Subcutaneous tumor models are an efficient and cost-effective choice for determining the tumor response to a new drug. Despite being straightforward, this approach displays several limitations including the complete loss of interaction between tumors cells and the bone microenvironment [16], resulting in a poor representative model of the disease. Another major limitation for the interpretation of data from these OS models is the biological difference of the vascular environment between xenografts grown in ectopic sites and the human tumor environment [17].

More recently, two alternative animal models, Patient Derived Xenografts (PDXs) and Genetically Engineered Mouse Models (GEMMs), were developed. PDX models are obtained by the direct engrafting of human biopsies in immunodeficient mice as a mean to reproduce the clinical situation of each tumor [18,19]. The more recent GEMMs follow the principle of activating or inactivating specific cancer-associated genes in immunocompetent mice, such as the tumor suppressors Tp53 and specific mutations mimicking Li–Fraumeni syndrome, which have been correlated with the etiology of OS [20]. However, both models suffer from significant limitations: PDXs models are characterized by a highly variable success rate (from 20% to 100%) correlated with the nature and availability of the original biopsy, whereas the development and validation of GEMMs models is time-consuming, laborious, and expensive.

The orthotopic model is an attractive alternative approach that overcomes these restraints. It is obtained either by injecting tumor cells paratibially or directly into the medullar channel, allowing the tumor growth at its innate site [16,21].

In the present work, we describe the procedure used to generate a tibial orthotopic OS model to test the anti-tumor effect of PTX-PDT combined treatment delivered within keratin nanoparticles. We then systematically show how we established the treatment key parameters, such as the design of the light-delivery device to induce PDT, the nanoparticle dose, the timeline regimen, and the irradiation settings. The OS growth inhibition is finally evaluated via histological examination coupled with imaging quantification analyses.

## 2. Materials and Methods

### 2.1. Reagents

Mc Coy’s medium, Dulbecco’s modified Phosphate buffer solution (D-PBS), TryPLE select, Fetal Bovine Serum (FBS), GlutaMAX, penicillin/streptomycin (P/S), TUNEL assay kit, Hoechst 33342, and ProLong™ Diamond Antifade Mountant, were purchased by Life Technologies-Thermo Fisher Scientific (Waltham, MA, USA). 2,7-dichlorodihydrofluorescein diacetate (H_2_DCFDA) and 9,10-dimethylanthracene (DMA) were purchased by Merck Life Science S.r.l. (Darmstadt, Germany). Paclitaxel was purchased by TCI-Europe (Zwijndrecht, Belgium). Chlorin-e6 was purchased from Livchem Logistics GmbH (Frankfurt am Main, Germany).

### 2.2. PTX-Ce6@ker Synthesis and Characterization

The full description of PTX-Ce6@ker nanoparticles synthesis and characterization is reported in our previous work [10]. Briefly, Ker-Ce6 and pristine keratin were dissolved in PBS pH 7.4 to achieve a Ce6 concentration of 40 µg/mg_ker_ and a keratin concentration of 5 mg/mL. Afterwards, the solution was sonicated for 30 min at 20 °C, and a solution of PTX (10% w_PTX_/w_ker_) in ethanol (10 mg/mL) was slowly added (0.3 mL/min) via syringe pump and under vigorous stirring (600 rpm). The solution containing PTX-Ce6@ker was then stirred for 1 h, analyzed by dynamic light scattering, and freeze dried.

The Reactive Oxygen Species (ROS) production was evaluated for PTX-Ce6@ker using the chemical probe 2,7-dichlorodihydrofluorescein diacetate (H_2_DCFDA) [22,23]. When ROS are present, the nonfluorescent molecule H_2_DCFDA is first hydrolyzed to 2,7- dichlorodihydrofluorescein (H_2_DCF) and then oxidized to the highly fluorescent species DCF. H_2_DCFDA was dissolved in methanol to form a 1.1 mM solution. Two mL of NaOH (0.01 M) were then added to 500 µL of this solution and stirred for 30 min at room temperature. Afterwards, 10 mL of phosphate buffer (pH = 7.4) were added to obtain the final ROS probe solution.

Samples were prepared as follows: 100 µL PTX-Ce6@ker diluted in water (2.5 mg/mL) were added to a cuvette containing 182 µL of water, 500 µL of phosphate buffer, and 218 µL of ROS probe (Ce6_final_ = 16.25 µg/mL). To excite the Ce6 photosensitizer present in the solution we used a white 320–700 nm emission wavelength tungsten lamp (300 W, light intensity 45 mW/cm^2^ at 670 nm, Phillips, Bologna, Italy). The light was positioned at a distance of 40 cm from the cuvette, and the absorption spectra were recorded at different time points with a Cary 100 UV–Vis spectrophotometer (Agilent Technologies, Milan, Italy) reading the absorbance at 500 nm.

Singlet oxygen (^1^O_2_) generation was determined by using the chemical probe 9,10-dimethylanthracene (DMA) [23]. Quartz cells (0.75 mL) with a 1 cm path length and containing 50 µL of PTX-Ce6@ker (0.625 mg/mL) and 650 µL of DMA in dimethylformamide (Ce6_final_ = 3 µg/mL) were irradiated with a white 320–700 nm emission wavelength tungsten lamp (300 W, light intensity 45 mW/cm^2^ at 670 nm, Phillips, Bologna, Italy) positioned at 20 cm from the cuvette and the absorption spectra were recorded at different time points with a Cary 100 UV–Vis spectrophotometer (Agilent Technologies, Milan, Italy) reading the absorbance at 378 nm.

### 2.3. Transmission Electron Microscopy (TEM) 

The morphology of keratin-based nanoparticles was analyzed by transmission electron microscopy (TEM). Briefly, PTX-Ce6@ker nanoparticles (0.1 mg/mL) were dispensed as a drop on a carbon-coated nickel grid and after 20 min any excess of the solution was absorbed by filter paper. The nanoformulation was subsequently observed with a Jeol Jem-1011 transmission electron microscope (Jeol Jem, Peabody, MA, USA). TEM images were then processed to measure the diameter of keratin-based nanoparticles (n = 319) for particle size analysis [24], which was carried out by fitting experimental data to a log-normal distribution function using the OriginPro 9 software (OriginLab Corporation; Northampton, MA, USA). Additional details are reported in the Supplementary Material (Table S1).

### 2.4. PTX Release Kinetics

The release kinetic of PTX from PTX-Ce6@ker nanoparticles was evaluated by equilibrium dialysis and HPLC with UV detection (HPLC-UV). Freeze-dried PTX-Ce6@ker nanoparticles (containing PTX 13%, *w*/*w*) were suspended in dialysis buffer ([PBS, pH 7.4]/ethanol 75:25, *v*/*v*) at a concentration of 375 µg/mL. The PTX-Ce6@ker samples (3 mL) were then loaded into a Pur-A-Lyzer Maxi dialysis tube (molecular weight cut-off: 12 kDa; Sigma-Aldrich, Milan, Italy) and dialyzed against 18 mL of dialysis buffer. Dialysis was carried out at controlled temperature (37 °C) over 29 h under gentle agitation using a M 200-TBP incubator (MPM Instruments, Bernareggio, Italy) and a Vibrax VXR S17 orbital shaker (IKA, Staufen, Germany). One-hundred-and-fifty-microliter aliquots were sampled from the release medium at defined dialysis times (0.5, 1, 1.5, 2, 2.5, 3, 4.5, 6, 7.5, 9, 21.5, 24, 26.5, and 29 h) and promptly replaced with an equal volume of fresh dialysis buffer.

All aliquots from dialysis experiments were then analyzed by HPLC-UV (n = 3), which was performed on a Nexera XR UHPLC system (Shimadzu, Milan, Italy) equipped with a LC-40D XR pump, a SIL-40C XR autosampler, a DGU-405 degassing unit, a CTO-40S column oven, an SPD-M40 photodiode array detector, and a Kinetex C18 column (5 µm, 100 Å, 150 × 4.6 mm; Phenomenex, Castel Maggiore, Italy). The analysis was carried out in isocratic conditions using a [trifluoroacetic acid 0.1% in acetonitrile, *v*/*v*]/[trifluoroacetic acid 0.1% in water, *v*/*v*] mixture (55:45, *v*/*v*) as mobile phase at a constant flow rate of 1.0 mL/min, a column oven temperature of 40 °C, a detection wavelength of 228 nm, an injection volume of 10 µL, and an autosampler temperature of 10 °C.

The quantification of released PTX was performed by calibration with six standard samples of PTX (0.1–25 µg/mL), prepared by dilution of a 10 mg/mL stock solution in dimethyl sulfoxide with dialysis buffer. The calibration curve was then derived by linear regression on HPLC peak areas for PTX ($t_{R}$ = 3.6 min) as a function of concentration; details are reported in the Supplementary Material (Table S2). PTX release was determined as the fraction of released compound ($f_{\mathrm{PTX}}$) relative to the total PTX content of the dialyzed sample (146.2 µg), which was determined from the cumulative peak area of PTX and its main degradation product 7-epipaclitaxel ($t_{R}$ = 5.3 min), assuming that its UV absorption can be approximated to that of PTX. Finally, the cumulative Weibull distribution function [25] was used to describe the full kinetic profile of PTX release from PTX-Ce6@ker nanoparticles, while the Korsmeyer–Peppas model [26] was applied to early-stage release data ($f_{\mathrm{PTX}}$ < 60%, *w*/*w*) to evaluate the release mechanism. Non-linear regression analysis using these models was performed with the OriginPro 9 software (OriginLab Corporation; Northampton, MA, USA); details are reported in the Supplementary Material (Tables S3 and S4).

### 2.5. Cell Culture

Saos-2 (HTB-85) cell line was purchased from ATCC (Manassas, VA, USA). Cells were cultured in McCoy’s medium containing 15% of FBS, 1% GlutaMAX, and 50 U/mL penicillin/streptomycin, at 37 °C in a humidified atmosphere of 5% CO_2_ air. Medium was changed twice a week and, when cells reached 70–80% of confluence, they were splitted for further passaging.

### 2.6. Orthotopic OS Model

For intratibial injections, Saos-2 cells were cultured up to 70–80% of confluency and harvested the day of cells inoculation. Cells were counted with Countess FL automatic counter (Life Technologies-Thermo Fisher Scientific, Waltham, MA, USA) and 5 or 10×10^6^ cells were resuspended in 50 μL-inoculation volume of D-PBS. 

Four-week-old male nude mice (BALB/c, nu/nu; Envigo RMS S.r.l., Azzida San Pietro Natisone, Udine, Italia) were housed according to D.L.vo 26/2014, directive 2010/63/EU, for the protection of animals used for scientific purposes, and according to the 2007/526/EC recommendation for the accommodation and care of animals used for experimental and other scientific purposes. All animal operations were approved by the local Ethical Committee (0024712/2015, 9 July 2015) and by the Animal Welfare Body (30 July 2015) of IRCCS Istituto Ortopedico Rizzoli and authorized by the Italian Ministry of Health (1271/2015-PR, 15 December 2015).

Fifty-two immunodeficient BALB/c Nu/Nu mice were used for the study: 10 mice for the model set up, 15 mice for the regimen set up and 27 mice for the efficacy study. Cells inoculation, nanoformulation delivery, and PDT were performed in all animals under sedation with general anesthesia in a plexiglass box. The anesthesia was maintained with a mixture of O_2_/air and isoflurane (2–3%) in spontaneous ventilation using a facial mask and keeping the animals on a warm pad to avoid hypothermia during the procedures.

For OS cells inoculation, the knee of each nude mice was bended beyond 90° to fa-cilitate the introduction of a 18 G needle aligned perpendicularly to the tibial plate to reach the medullar channel. This helped to create a canal to introduce a Hamilton syringe loaded with a 26 G needle to inject the cells [27]. Cells inoculation was performed on both tibias, and the tumor growth was assessed weekly with dorsal-ventral X-ray imaging under general gaseous anesthesia. Animal wellbeing was monitored daily and by weekly weight measurements.

### 2.7. PDT Device

The LED device was designed and manufactured in-house to perform in vivo studies. The prototype consists of two flexible arms equipped with LED lights (LZ4-00R208 Deep Red, 660 ± 2 nm; 6.6 W; light intensity 93.3 mW/cm^2^ at 670 nm, Mouser Electronics, Mansfield, TX, USA) topped with two heating dissipators and two black cylinders to focus the light on the leg’s area and spare the rest of the body.

### 2.8. Histological Analyses

All the animals were sedated with intramuscular Rompun^®^ (xylazine) injection and then euthanized by intracardiac Tanax^®^ (MSD Animal Health S.r.l., Segrate, Milano, Italy). Tibia explants were collected, fixed in 4% in paraformaldehyde for 24 h, decalcified in 4% EDTA solution for 7 days at RT, changing the solution after 3 days, included in paraffin, and processed.

Paraffin sections of 5 μm thickness obtained by cutting with a microtome along the longitudinal plane to include the entire tibia length, were mounted onto Poly-lysine glass slides and stained with Haematoxylin & Eosin (H&E) according to the anatomical his-topathology service protocol used (Istituto Ortopedico Rizzoli). H&E slides of all tumors were reviewed with the help of a human pathologist (Dr. Marco Gambarotti), and the morphological diagnosis was confirmed based on cellularity, nuclear pleomorphism, and presence of necrosis as universal and standardized criteria defining malignancy.

TUNEL assay (Click-iT Tunel colorimetric IHC detection kit) was performed according to manufacturer’s protocol with the following minor modifications: proteinase K incubation was settled at 20 min at RT and the DAB reaction mixture incubation was defined at 15 min.

For immunohistochemical analysis, unstained sections were heat-treated at 60 °C for 20 min, deparaffinized, and immunostained on a Ventana BenchMark following the manufacturer’s guidelines (Ventana Medical Systems, Tucson, AZ, USA). Immunostaining was performed using the K_i_-67 rabbit monoclonal primary antibody (clone 30-9; Ventana). Avidin–biotin complex peroxidase assays were performed to analyze the expression of K_i_-67. All images were acquired using Aperio Digital Pathology Slide Scanner (Leica Biosystems, Milano, Italy) and the obtained images were analyzed using QuPath software, an open-source software for digital pathology image analysis [28].

Tumor area quantification was performed by manual selection from two operators in every H&E image. On the same images, the cells quantification was performed by se-lecting five different ROIs of 10^5^ μm^2^ area within the entire tumor area region, then nuclei were manually counted using QuPath (cell counting tool). The obtained results were averaged and plotted. As for H&E staining, K_i_-67 and TUNEL positive cells were quantified defining four different ROIs of 5 × 10^4^ μm^2^ area within the entire tumor area region previously identified, and the data were averaged and plotted.

### 2.9. Confocal Laser Scanning Microscopy and Fluorescent Lifetime Imaging

Paraffin embedded slices were first rehydrated and then incubated for 60 min with 10% normal goat serum (Thermo Fisher Scientific) diluted in PBT (blocking solution). After several washes with D-PBS, slices were incubated with Hoechst 33342 (Thermo Fisher Scientific) diluted 1:2000 in D-PBS for 10 min. After several washes with D-PBS, samples were mounted with ProLong™ Diamond Antifade Mountant (Life Technologies). Fluorescence confocal imaging was performed on an inverted Nikon Ti-E microscope (Nikon Co., Tokio, Japan). The confocal fluorescence microscope Nikon A1 is equipped with an Argon ion CW laser, a 640 nm CW diode laser, 405 nm and 640 nm pulsed/CW diode lasers (PicoQuant GmbH, Berlin, Germany). Images were collected using either a Nikon Plan Apo VC 20X air objective with NA 0.8 or a Nikon Plan Apo VC 60X oil immersion objective with NA 1.40. Filters were set to register the fluorescence in the 460–500 nm, 510–540 nm, 555–615 nm, and 665–735 nm ranges. Nikon A1 spectral module with a precisely corrected 32-PMT array detector is used for spectral imaging. Wavelength resolution was set to 6 nm per PMT.

Fluorescence lifetime imaging (FLIM) was performed exciting with the pulsed 405 nm and 640 nm diode laser and collecting photons at 655/40 nm and 716/40 nm with integrated PicoHarp 300 electronics (PicoQuant GmbH, Berlin, Germany) for time-correlated single photon counting (TCSPC) measurements. Histograms of collected photons consist in 1600 channels each with 16 ps width. A single-photon avalanche diode detector equipped with a bandpass filter was used as detector. The repetition rate of the pulsed excitation was 40 MHz. The instrument response function of the system is approximately 220 ps. The fluorescence decay fit was performed on the histogram calculated for pixels with a number of photons above threshold in the tissue sample image. The fluorescence decay profile was analyzed with a least-squares method, using bi- or tri-exponential decay functions provided by Picoquant SymPhoTime software. Calculated Instrumental Response Function was used for reconvolution. The average fluorescence lifetime image was calculated fixing the lifetimes obtained from the analysis of the histogram of the region of interest while the software calculates the preexponential factors for each pixel.

FLIM exciting at 405 nm and collecting photons in the 635–675 nm range afforded better results compared to excitation at 640 nm collecting emission in the 700–740 nm range as scattering is contributing strongly to the fluorescence collected in the 700–740 nm range. The contribution of Hoechst excitation at 405 nm is negligible at the 635–675 nm emission range and can be separated from Ce6 emission. Hoechst has an average fluorescence lifetime of 2.1 ns (measured in the 460–500 nm range) in the tissues.

Ce6 fluorescence decays in solution were measured in air-equilibrated water solution for excitation at 637 nm (Hamamatsu pulsed laser with 1 MHz repetition rate) using a TCSPC system (IBH Consultants Ltd., Glasgow, UK) with a resolution of 55 ps per channel. Photons were detected in right angle configuration at 690 nm with a cut-off filter. Fluorescence decay profiles were analyzed with a least-squares method, using multi-exponential decay functions (Equation (2)) and deconvolution of the instrumental response function. The software package was provided by IBH Consultants Ltd.

(1) $$\mathrm{The}\;\mathrm{fitting}\;\mathrm{function}\;\mathrm{used}\;\mathrm{is}:\;I\left(t\right)=\;b\;+{\;\Sigma }_{j}a_{j}e^{\left(-t/\tau_{j}\right)}$$

The fractional intensity and the average fluorescence lifetime are calculated according to the following equations:

(2) $$f_{i\;}=a_{i}\tau_{i}/\Sigma_{j}a_{j}\tau_{j}\;\;\;\tau_{\mathrm{av}}={\;\Sigma }_{j}{\;f}_{j}\tau_{j}$$

### 2.10. Statistical Analysis

The data shown in this study are expressed as mean ± SD. All statistical analyses were performed with GraphPad Prism 6 software (GraphPad; San Diego, CA, USA) using One-way ANOVA followed by Tukey’s multiple comparisons test. Significance was represented as follow: * = *p* ≤ 0.05; ** = *p* ≤ 0.01; *** = *p* ≤ 0.001; not significant (n.s.) = *p* > 0.05.

## 3. Results and Discussion

### 3.1. Chemical and Physical Characterizations of PTX-Ce6@ker Nanoformulation

**Figure 1:**
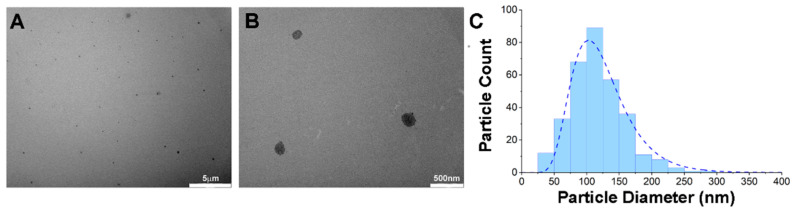
Physical characterizations of PTX-Ce6@ker nanoformulation. (**A**,**B**) Representative Transmission electron microscopy (TEM) micrographs of PTX-Ce6@ker nanoparticles performed at a final concentration of 0.1 mg/mL (5 μm and 500 nm scale bar, respectively). (**C**) The graph shows the diameter distribution of keratin-based nanoparticles determined by analysis of TEM images (n = 319).

Synthesis and characterizations of dual-loaded keratin nanoparticles are reported in our previous manuscript [10]. For the in vivo experiments, a larger scale production was required, and the nanoparticles were synthetized to contain a PTX and Ce6 concentration of respectively 130 μg and 59.16 μg per mg of keratin nanoparticles. To confirm the quality and reproducibility of the overall procedure, we tested the polydispersity index in D-PBS at 37 °C and the data confirmed a value between 0.15–0.18 as observed in previous preparations [10]. The physical characterizations of nanoformulation were performed by measuring the particles’ diameter with TEM imaging (Figure 1A,B and Figure S1), and results show that the nanoformulation displays an average diameter of 120 nm (Figure 1C).

**Figure 2:**
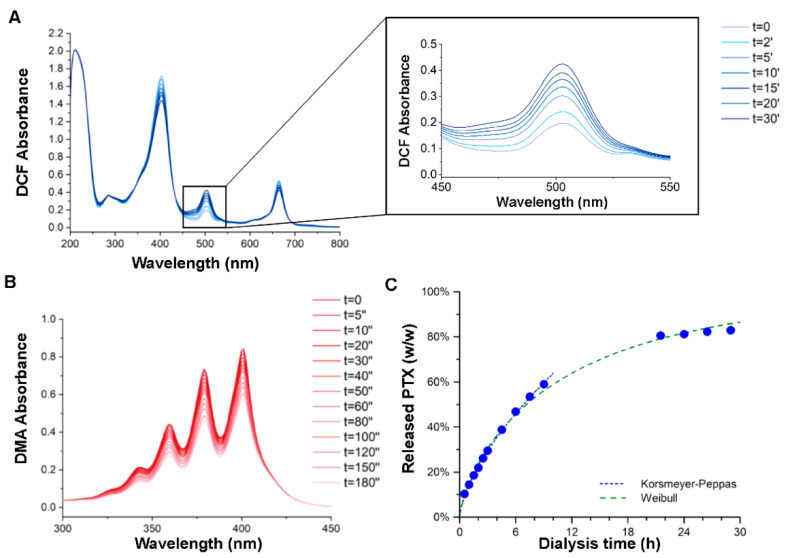
Chemical characterizations of PTX-Ce6@ker nanoformulation. (**A**,**B**) The graphs show the absorption spectra reflecting ROS and ^1^O_2_ production, respectively, measured at different irradiation times, expressed as minutes for ROS (‘) and seconds (‘’) for ^1^O_2_. (**C**) Release kinetics of PTX from PTX-Ce6@ker nanoparticles, as determined by equilibrium dialysis at 37 °C in (PBS, pH 7.4)/ethanol (75:25, *v*/*v*) and HPLC-UV analysis. Korsmeyer–Peppas model: $y=0.1461x^{0.6406}$ (R^2^ = 0.9962); Weibull model: $y=1-e^{-0.1621x^{0.7898}}$ (R^2^ = 0.9987).

The chemical characterization was then performed to establish the generation of reactive oxygen species (ROS) and singlet oxygen (^1^O_2_) upon light stimulation, while the PTX release was measured via HPLC analysis (Figure 2).

ROS generation from Ce6 loaded into PTX-Ce6@ker nanoparticles was calculated by measuring the increase of 2,7-dichlorofluorescein (DCF) absorption peak at 500 nm. The non-fluorescent molecule H_2_DCFDA is first hydrolyzed to H_2_DCF and then oxidized to the highly fluorescent DCF only in the presence of ROS (see baseline in Figure S2A). For this assay, a solution of PTX-Ce6@ker and ROS probe (see Section 2.2 for details) was irradiated with a white tungsten lamp (300 W) for different time intervals and the DCF absorbance was recorded straight after the irradiation. The absorbance peak at 500 nm increased with the irradiation time (Figure 2A), indicating that PTX-Ce6@ker nanoparticles generate ROS in a light dose-dependent manner.

Among ROS, ^1^O_2_ is strictly involved in the oxidative cellular damages induced by PDT. Based on this, its production by PTX-Ce6@ker was investigated by recording the decrease of 9,10-dimethylanthracene (DMA) absorption peak at 378 nm. DMA is converted to its non-fluorescent endoperoxide form only in the presence of ^1^O_2_, therefore, as expected, the solution containing the DMA probe did not show any significant decrease of the 378 nm peak upon irradiation (see baseline in Figure S1). Conversely, the PTX-Ce6@ker solution in the presence of DMA produced a gradual decrease of absorbance at 378 nm, proportionally to the increase of the irradiation time (Figure 2B). Thus, even when loaded into keratin nanoparticles, Ce6 can produce both ROS and singlet oxygen in a light dose-dependent manner.

Equilibrium dialysis was carried out at 37 °C in (PBS, pH 7.4)/ethanol (75:25, *v*/*v*) to evaluate the release of PTX from PTX-Ce6@ker nanoparticles; dialysis conditions were finely tuned to avoid PTX precipitation inside the dialysis tube upon release [29]. High-performance liquid chromatography (HPLC) analysis on the release medium (Figure 2C, Table S3) highlighted that, according to the Korsmeyer–Peppas model, PTX-Ce6@ker nanoparticles follow a non-Fickian mechanism of PTX release driven by both diffusion and swelling of the keratin matrix at similar rates (diffusional exponent $n_{P}$ = 0.641) [30], reaching 80% of total PTX release within the first 24 h in the dialysis settings. The overall kinetic profile described by the Weibull model yielded a half-time of 7.1 h for the release of the chemotherapeutic agent (Table S4).

### 3.2. Orthotopic Osteosarcoma Mouse model Set Up

For the preclinical in vivo evaluation of the chemo-releasing and photoactive keratin nanoparticles, we generated an orthotopic osteosarcoma murine model using human-derived OS cells (Saos-2). Two different dosages of Saos-2 cells (5 × 10^6^ and 10 × 10^6^ per injection/tibia) were initially tested to verify the capacity to induce tumor formation and to establish the most efficient dosage to achieve the highest percentage of engrafting rate. The shirring stress due to the passage of the cells through the syringe needle used for the inoculation in the mouse tibia was evaluated by measuring the cell viability in vitro. A 26 G needle was selected, since it can guarantee high cell viability after the extrusion of 5 × 10^6^ cells (96.5% viability pre- vs. 87% post-injection), and of 10 × 10^6^ cells (96.9% viability pre- vs. 88% post-injection).

**Figure 3:**
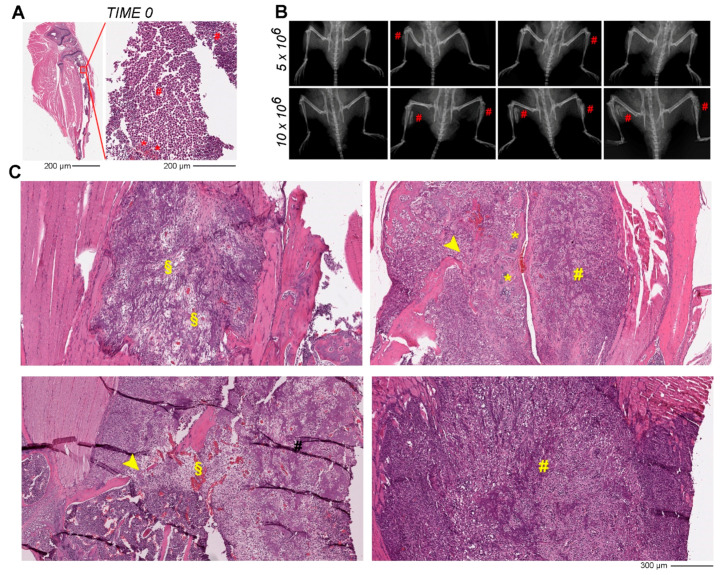
Preclinical Osteosarcoma mouse model set up. (**A**) Representative paraffin section stained with H&E showing the entire tibia right after the inoculation of Saos-2 cells (TIME 0). The magnification of the red squared area is shown in the right panel. The red hash marks (#) indicate the OS tumor cells, while the red asterisks (*) the bone marrow cells. (**B**) Representative X-ray imaging of four animals from the 5 × 10^6^ Saos-2 group at week 6 from initial inoculation, and of four animals from the 10 × 10^6^ Saos-2 group at week 8 from initial inoculation. The red hash marks (#) point to the detectable tumor mass. (**C**) Representative paraffin sections stained with H&E of 10 × 10^6^ Saos-2 inoculation groups at 8 weeks. The § indicates the necrotic area, the hash marks (#) indicate new bone matrix, the head-arrows indicate the bone trabeculae damage due to tumor growth, while the asterisks the chondroid matrix.

The OS cells were injected into both tibias of the animals, and the tumor growth was monitored weekly via X-ray imaging (Figure S3). The injection site, as verified with histology and H&E staining on euthanized animals right after the inoculation, fits in proximity or inside the bone marrow canal (Figure 3A). In the 5 × 10^6^ Saos-2 group, a traceable mass was present in one tibia (2 animals out of 4) 6 weeks after the initial inoculation, giving an engrafting rate of 25% (Figure 3B). The engrafting rate increased up to 75% when injecting a double amount of tumor cells (10 × 10^6^). X-ray imaging over time clearly revealed visible tumor masses in both tibias in three animals out of four starting from week 4 after the initial inoculation in the 10 × 10^6^ Saos-2 group (Figure 3B and Figure S3). The tumor, as evidenced by H&E staining, develops within the medullae canal, lysing the trabecular bone with evident signs of extravasation and invasion into the muscle areas (Figure 3C, yellow head-arrows). It is characterized by the production of new bone matrix (Figure 3C, yellow hash marks) combined with compromised periosteum due to the lytic effect exhibited by the OS tumor cells, particularly evident in the 10 × 10^6^ Saos-2 group. The tumor also presented areas of chondroid matrix formed in the midst of neoplastic cells (Figure 3C, yellow asterisks) and necrotic regions particularly evident in the biggest lesions (Figure 3C, yellow §). These histological features are well reported in previous murine OS models [16] and they recapitulate the hallmarks of physiological OS lesions in affected patients [31,32].

The histological analysis revealed a limitation of the X-ray imaging since in some cases radiographic pictures did not show evident signs of tumor formation inside the medullar channel, where the resolution of the X-ray is not sensitive enough to distinguish the tumor mass, making its detection difficult and the corresponding quantification of the tumor area very challenging. Imaging modalities such as radiography have the ability to characterize tumors on the basis of information such as sclerotic changes, osteolysis, and periosteal reactions in diagnoses of bone lesions, or calcification and skeletal invasion in soft tissue lesions [33]. Moreover, in the treatment of malignant bone tumors, the chemotherapeutic effect can be qualitatively assessed by sclerotic changes and cortical bone remodeling with radiography. Nevertheless, quantitative and objective assessment of the chemotherapeutic effect is difficult [34], therefore, the histopathological analysis is still the preferential way to assess tumor formation and to evaluate tumor reduction in preclinical animal models [35].

The 10 × 10^6^ Saos-2 group was then chosen as the OS model to test the treatment regimen and the efficacy of the nanoformulation-based therapy. For the large study group, despite some variability observed in the size and dimension of the tumor, a total of 60% tumor engrafting rate was achieved using 10 × 10^6^ Saos-2 cells inoculated in both tibias.

### 3.3. Treatment Regime Set Up

PDT is known to induce early and late side effects when dosages of photosensitizers or light are not well calibrated. Erythema, pain, burns, edema, itching, desquamation, and pustular formation, often in association with each other, are frequently observed during the exposure to the light source and in the hours/days immediately after the therapy. In particular, pain is a clinically relevant short-term complication that also reduces long-term patient satisfaction [36]. Predictors of pain intensity and aspects of pain management during topical PDT using photosensitizers already approved for clinical usage [37] help in defining the proper dosages and light treatment timeline to avoid pain and other discomforting or damaging side effects.

Taking into consideration all these aspects, the next phase of the preclinical study focused on establishing several key treatment parameters, such as: the design of the light-delivery device, the nanoformulation dosage, and the treatment schedule.

#### 3.3.1. Light-Delivery Device

To perform PDT, we designed a custom-made device capable of exciting Ce6 and simultaneously irradiating both mouse tibias, sparing other body regions.

**Figure 4:**
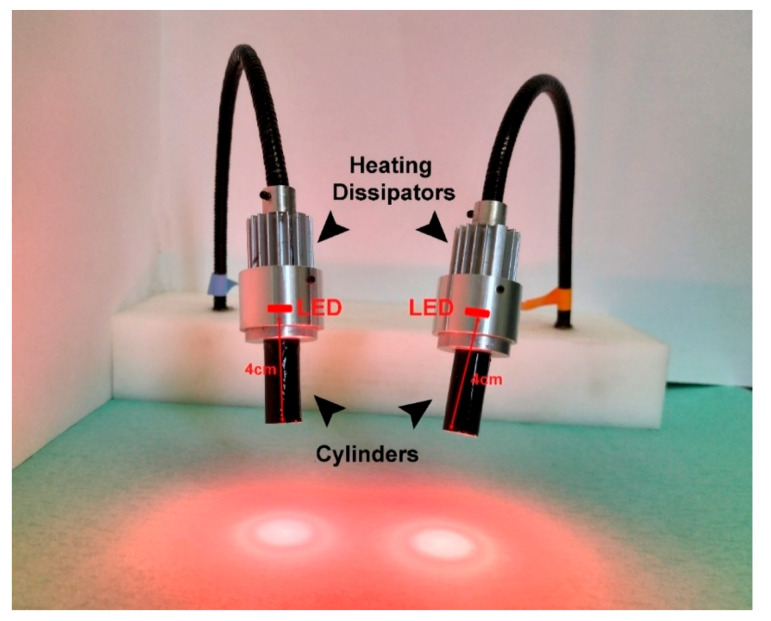
Device prototyped for PDT in the in vivo experimentation. Two 660 ± 2 nm wavelength LED lights were assembled on two distinct flexible arms to focus the light directly on the treated area. To converge the irradiation, two cylinders (4 cm × 1 cm) were added on top of the light source to focus the light area over the animal’s tibia, and two heat dissipators were mounted to prevent excessive thermal surcharge on the lights.

Therefore, we manufactured a prototype (Figure 4) characterized by two flexible arms equipped with LED lights (660 ± 2 nm) topped with two heating dissipators and ended with two black cylinders to focus the light on the tibias area. To find the best light irradiation conditions, the stimulation was kept continuous for 15 min. Considering that the light dose administered to the tibia during the irradiation is significantly dependent on the distance between the light source and the target tissue, we performed several tests on mice inoculated with D-PBS (data not shown, control group) varying the distance between the cylinder end and the animal skin (direct contact, 1 or 2 cm distance). The light dose was 190 J/cm^2^ when in direct contact with the skin, 84 J/cm^2^ keeping 1 cm of distance, and 49 J/cm^2^ at 2 cm distance.

The irradiation performed at the shortest distance caused burning on the skin, while this side effect was not observed in the other two tested conditions. The 2 cm distance caused spreading of the light over and above the tibia area. Therefore, to avoid any injuries caused by the detrimental thermal effect and to concentrate the light only on the tibia area, the animal experimentation was performed by keeping the LED light at 1 cm distance from the bottom of the cylinders to the surface of the mouse leg with a total fluence of 84 J/cm^2^.

The penetration of the light inside the bone tissue was validated based on our previous study performed on a prostate tumor model. In this study the tumor was induced intramuscularly in the mouse femur and the efficacy of a nanoformulation (FNP) carrying phthalocyanine (Ptl) as a photosensitizer to induce PDT (Ptl@FNP) was demonstrated. When stimulated with 680 nm wavelength LED light at 2 cm distance from the leg, the light was able to penetrate the skin and the muscle tissue, to generate ROS from Ptl@FNP nanoparticles and to significantly reduce tumor growth [38]. Moreover, PDT using 635 nm wavelength laser light as an external irradiation source has been shown to be able to reach the bone lesion in a primary canine bone tumor model [39] and induce massive cell death 48 h post irradiation.

#### 3.3.2. Nanoformulation Dosage and Treatment’s Schedule

After setting the irradiation conditions, we established the dose of the PTX-Ce6@ker nanoformulation to be used for the treatments. We tested three dosages (Table 1) in nine animals (n = 3/dose). A total volume of 50 μL of nanoparticles, resuspended in water, were locally inoculated close to the tumor area in two distinct sites with 25 μL volume per injection.

**Figure 5:**
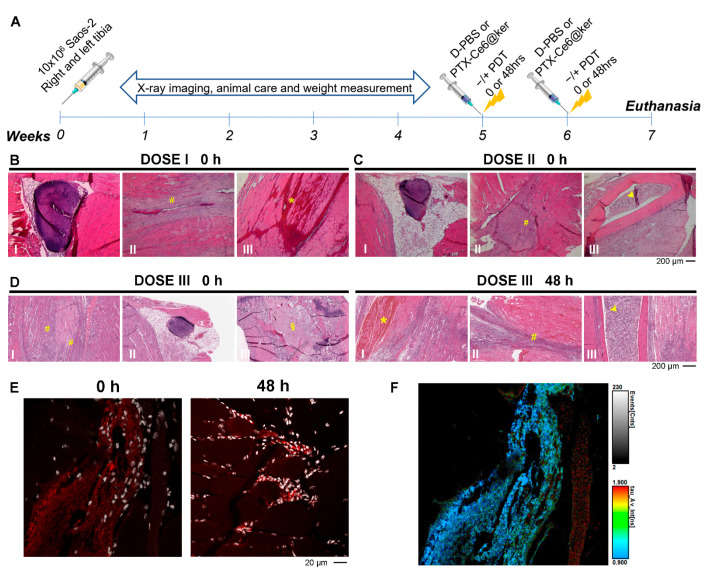
Treatment regime set up: nanoformulation dosage and treatment’s schedule. (**A**) Timeline of the treatment regimen. (**B**–**D**) Histological evaluation of tumor tissue after treatments. Representative brightfield images of paraffin sections stained with H&E from samples treated with DOSE I (**B**), DOSE II (**C**) and irradiated immediately after (0 h), and of DOSE III (**D**) irradiated at 0 h and after 48 h. (**E**) Representative confocal images of samples from mouse treated with DOSE III and euthanised immediately (0 h) or 48 h after treatment. The red signal corresponds to the Ce6 molecule, while nuclei are shown in white. (**F**) Representative figure of FLIM analysis showing the Ce6 signal distribution in the tissue area right after PTX-Ce6@ker inoculation (time 0 h) with the colour scale indicating the average lifetime. The blue regions with τ_av_ of around 1 ns are indicative of Ce6, while the red-coloured regions, characterized by a τ_av_ of > 2 ns, are indicative of autofluorescence and Hoechst staining in the nuclei.

The treatment regime was planned as two cycles of nanoparticles injection followed by PDT (84 J/cm^2^) or no light irradiation, performed respectively at week 5 and 6 after the initial OS tumor cells inoculation (Figure 5A). The sacrifice was scheduled at week 7 and it was followed by histological processing of the tibias.

In the dose I group (97.5 µg/tibia PTX), only one mouse out of three tolerated the treatment, while the other two animals showed skin lesions after the first cycle of treatment and were euthanized. The histological analyses revealed that the treatment immediately followed by irradiation (0 h) induced the inflammation of the popliteal lymph node (Figure 5B-I) and a massive immune system response that led to muscle damage (Figure 5B-II, yellow hash marks) and, in some cases, complete muscle atrophy (data not shown). Moreover, significant histological signs of oedema in muscles were observed (Figure 5B-III, yellow asterisks). We concluded that dose I was unsuitable for treatment, and we then investigated doses II and III, corresponding to 75% and 50% of dose I, respectively. In animals treated with dose II, no inflammation of the popliteal lymph node was observed (Figure 5C-I), but a strong immune response was evident in all animals treated with nanoparticles followed by light irradiation (Figure 5C-II, yellow hash marks). Moreover, the animals developed an important damage to the bone marrow (Figure 5C-III, yellow head arrow), and only two out of three mice tolerated the full treatment. On the contrary, in the dose III group, all three animals survived, and the histological analyses revealed a massive inflammatory response immediately after the treatment (Figure 5D-I, 0 h, yellow hash marks), without affecting the popliteal lymph node (Figure 5D-II, 0 h) and the necrosis of tumor cells inside the tibia’s head (Figure 5D-III, 0 h, yellow §).

To select the irradiation timing with dose III, mice were exposed to light immediately (0 h) or 48 h after the injection of PTX-Ce6@ker nanoformulation. Clinical observations revealed that mice exposure to light 48 h after PTX-Ce6@ker injection led to oedema formation on the skin in two out of five mice, as confirmed by the massive presence of erythrocytes in the muscle area (Figure 5D-I, 48 h, yellow asterisks).

Two mice were euthanized because of exhibiting signs of physical distress, probably due to the massive immune cells’ infiltration in the muscle (Figure 5D-II, 48 h, yellow hash marks) and to the significant damage of the bone marrow (Figure 5D-III, 48 h, yellow head arrow). One animal over five tolerated the complete treatment. These side effects could be ascribed to the diffusion of the nanoformulation away from the initial site of injection (muscles surrounding the tumor), inducing skin hypersensitivity and, as consequence, burning effects, not compatible with multiple treatments. In addition, Ce6 molecule distribution is likely changing over 48 h in the tumor tissue, possibly impacting the PDT effect. To observe the photosensitizer biodistribution, confocal imaging exploiting Ce6 intrinsic fluorescence was performed on tissue samples of mice euthanised at time 0 after the nanoparticles inoculation (dose III) and after 48 h without being irradiated (Figure 5E). Spectral imaging as well as fluorescence-lifetime imaging (FLIM) were performed to investigate the fluorescence of chlorin e6, which strongly depends on its tissue distribution. Spectral imaging was performed upon excitation at 405 or 488 nm to discriminate Ce6 emission from the tissue autofluorescence and Hoechst fluorescence (Figure S4). At time 0, samples predominantly display areas where we can discern diffuse Ce6 emission in muscles, adipose areas, and, in a few regions, also inside the cells. After 48 h, samples show several cells presenting bright spots due to Ce6 emission. These cells are grouped in larger areas or diffused among the muscles (Figure 5E and Figure S5).

Because the fluorescence lifetime of a fluorophore is very sensitive to the local environment, FLIM was also performed (Figure 5F and Figure S6). The fluorescence lifetime of Ce6 in D-PBS is 3.8 ns while, when loaded on the keratin NPs, Ce6 shows lifetimes of 1.8 and 4.3 ns, indicating that Ce6 locates at least in two different environments within the NPs (Table S5). The shorter lifetime may be due to an electron transfer involving the protein or to Ce6 self-quenching. The FLIM analysis of tissues required a tri-exponential decay function, suggesting that Ce6 is localized in different environments. Moreover, the Ce6 average fluorescence lifetime in the tissue is in the 0.8–1.0 ns range, significantly shorter than that of Ce6@ker in solution, indicating that Ce6 may be, in part, no longer on the nanoparticles, independently of the inoculation time. To further corroborate this hypothesis, future studies will be focused on understanding the degradation rate of the keratin nanoparticles used as a delivery platform, and the corresponding release profile of the Ce6.

According to the above observations, dose III and irradiation at time 0 were selected as the most suitable parameters for evaluating the treatment efficacy.

### 3.4. Preclinical Evaluation of the Therapy Efficacy

**Figure 6:**
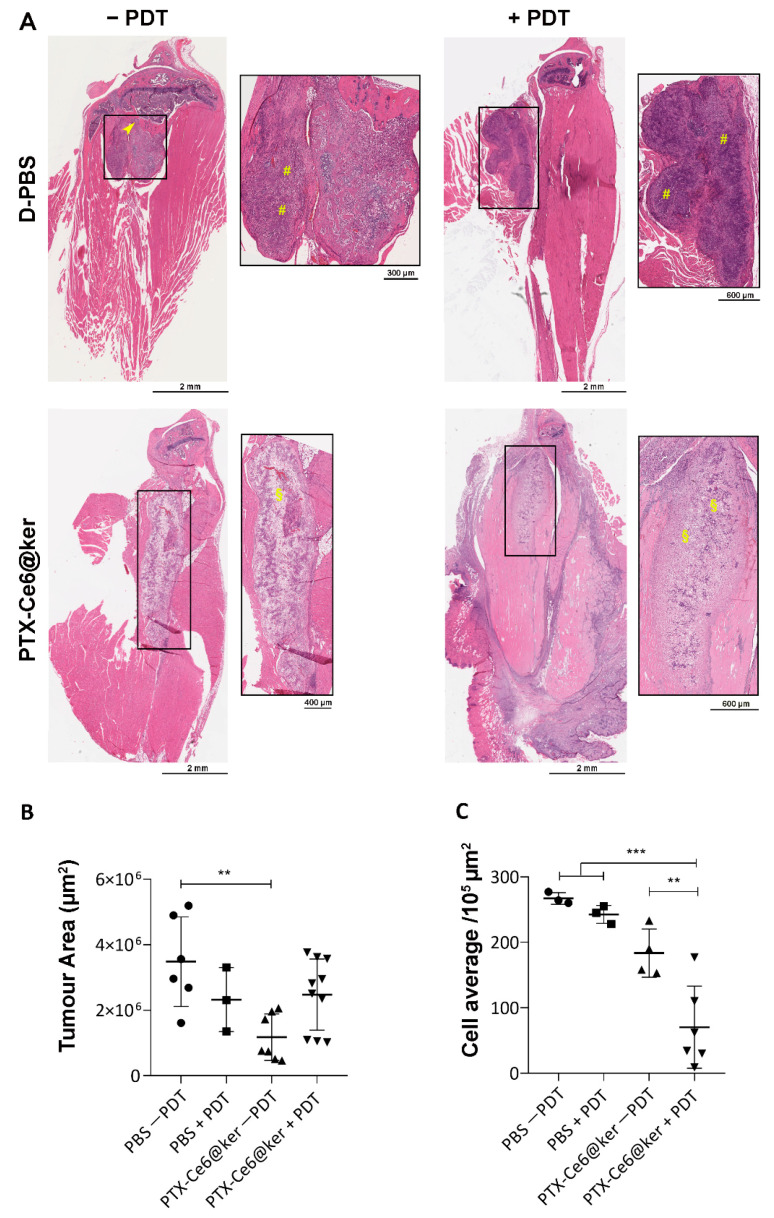
Preclinical evaluation of the therapy efficacy. (**A**) Representative H&E stained paraffin sections from tibia explants of animals from control groups (D-PBS −/+ PDT) and treatment groups (PTX-Ce6@ker −/+ PDT). Magnification of the black squared area is shown on the right side of the main panels. (**B**,**C**) The graphs show the quantification of tumor area and tumor cell number, respectively, performed with QuPath software. Data are expressed as the mean ± SD and analyzed using the One-way ANOVA test, and Tukey’s multiple comparison test as a post-test. Results were statistically significant at *p* values < 0.05 (** *p*-values< 0.01; *** *p*-values < 0.001).

**Figure 7:**
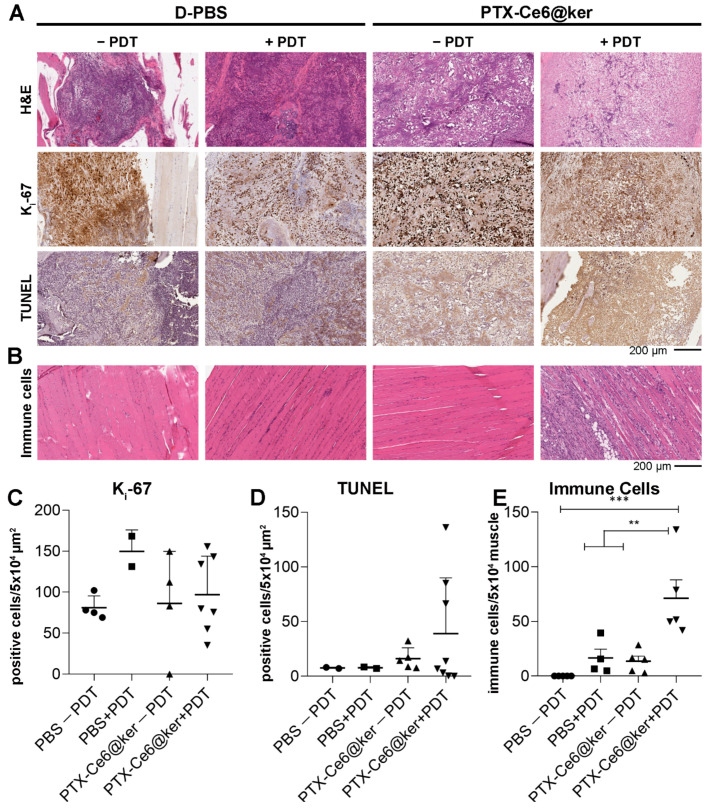
Preclinical evaluation of the therapy efficacy. (**A**) Representative paraffin sections from tibia explants of animals from control groups (D-PBS −/+ PDT) and treatment groups (PTX-Ce6@ker −/+ PDT) stained with the following markers: H&E, K_i_-67, and TUNEL. (**B**) Representative paraffin sections from muscle explants surrounding the tumor, stained with H&E. (**C**,**D**) The graphs show the quantification of K_i_-67 and TUNEL positive cells in four selected ROI/slices along the tumor tissue, respectively. (**E**) The graph shows the quantification of the immune cells in the muscle tissues after treatments. Data are expressed as the mean ± SD and analyzed using the one-way ANOVA test and Tukey’s multiple comparison test as a post-test. Results were statistically significant at *p* values < 0.05 (** *p* values < 0.01; *** *p* values < 0.001).

The effects due to chemotherapy and phototherapy were evaluated by comparing the PTX-Ce6@ker nanoformulation outcome in animals without (PTX effect only) and with (the combination of both PTX and PDT) light irradiation (84 J/cm^2^). At week 7, animals were euthanized and the tibias with all surrounding tissues were explanted and processed for histological analyses. The effects of the treatments were assessed with the following stainings: H&E for histopathological observation and quantification of tumor growth, K_i_-67 as a marker of tumour cells proliferation, and TUNEL assay as a readout of cells death (Figure 6 and Figure 7).

The histology of the control group confirmed that the tumor develops in the medullae canal, lysing the trabecular bone and invading the surrounding muscles (Figure 6A, head-arrow), and it is characterized by actively proliferating tumor cells (Figure 7A, K_i_-67) and the deposit of new bone matrix (Figure 6A, hask masks). The light irradiation on control animals (D-PBS + PDT) did not cause any sign of distress or skin burning, and the H&E staining did not reveal any significant change in terms of immune system activation (Figure 7B), increase of necrotic area within the tumour mass, reduction of tumor size, or damages to other tissues such as muscles and bone marrow. Taken together, these results highlight that the exposure to the light source itself is not detrimental for the animals. The treatment with PTX-Ce6@ker in the absence of light irradiation led to a significant reduction in tumour size as calculated via tumor area quantification (Figure 6B, See Section 2.8).

Significant areas of necrotic cells were also spotted (Figure 6A, §). The histology of animals treated with PTX-Ce6@ker followed by light irradiation revealed the presence of necrotic areas (Figure 6A, §), and the quantification of tumor area highlighted a 30% reduction compared to control group (Figure 6B). Moreover, the cells count performed on the histological sections further supported the inhibition of tumor cells viability in the animals treated with and without PDT (Figure 6C). The nanoformulation effectively released PTX, inducing a 32% reduction of viable cells without light, confirming the data obtained by the tumor area analysis. The light irradiation elicited a synergistic effect that decreased the number of viable tumor cells up to 78% and led to the formation of massive necrotic areas within the tumor with evident signs of immune cells reaction (Figure 6B,C and Figure 7E).

We then further analyzed the tibias explants using markers of proliferation and cell death (Figure 7). The images revealed an important reduction of actively proliferating tumor cells in the animals treated with the nanoformulation upon PDT (Figure 7A,C). The samples treated with PTX-Ce6@ker +PDT can be divided into two sub-groups, each characterized by a significant difference in the number of K_i_-67 positive cells. This result can be associated to the higher infiltration of immune cells which, being positive for K_i_-67 staining, are impairing the quantification of the tumor cells. The active role of immune system in mice treated with PTX-Ce6@ker is confirmed by the massive induction of the immune system reaction observed in the treatment groups (Figure 7B), which was significantly enhanced upon light irradiation (Figure 7E). The animals exposed to nanoparticles and irradiated showed an evident increase of the TUNEL positive cells compared to all other experimental groups (Figure 7D).

Overall, our data demonstrate the direct efficacy of the dual therapy on OS tumor cells growth and a significant inhibition of actively proliferating cells, together with a synergistic effect derived from the combination of PTX release and Ce6-PDT. While in our analyses we observed a significant reduction on the tumor area in the PTX non-PDT group, the light irradiation caused a massive inflammatory reaction coupled with the formation of edematous and necrotic areas. In fact, the significant activation of the immune system in some animals contributed to the development of edema and to a slight increase of the tumor area. Nevertheless, the cell count quantification confirmed that these regions contained fewer proliferating cells compared to untreated controls.

Besides causing direct cytotoxic effects on irradiated tumor cells, PDT is known to cause damage to the tumor vasculature and induce the release of pro-inflammatory molecules. Pre-clinical and clinical studies have already demonstrated that PDT can affect both the innate and adaptive arms of the immune system [40]. Immune stimulatory properties of PDT may increase its beneficial effects, giving the therapy wider potential to become more extensively used in clinical practice for the treatment of bone lesions as well. Moreover, mounting evidence highlights the crucial role of PDT in eliciting the immunogenic cell death (ICD) through ROS production. ICD triggers the release/exposure of damage-associated molecular partners (DAMPs) from dying cancer cells, thereby leading to the recruitment of antigen-presenting cells and restoring the host immune response [41]. Besides stimulating tumor-specific cytotoxic T-cells capable to destroy distant untreated cancer cells, PDT leads to the development of anti-tumor memory immunity that can potentially prevent cancer recurrence [42].

## 4. Conclusions

Keratin-based nanoparticles delivering a combination of photodynamic therapy and chemotherapy can represent a double punch in treating osteosarcoma tumor and provide significant advantages to overcome MDR and reduce systemic toxicity, which represent the major challenges in cancer care. The results reported in the present work show how, when injected directly within the tumor area, the PTX-Ce6@ker nanoformulation induces osteosarcoma cancer cells death, a massive activation of the immune system, and a synergistic effect deriving from the combination therapy. Future investigations will focus on determining the fate of the keratin nanoformulation post-treatment and the specific effect on signaling pathways involved in the immune cell’s response observed in treated animals.

Our findings provide the in vivo proof of concept for the next clinical development of a combined nanosystem for the treatment of osteosarcoma. The selective delivery of anti-cancer compounds gives us the opportunity to utilize drugs that have been previously discarded due to their high systemic toxicity. Once this treatment is proven effective in OS patients, this therapeutic approach may also be indicated to treat patients with challenging lesions derived from other type of bone tumors.

## Figures and Tables

**Table 1:** Summary of the tested dose of PTX in PTX-Ce6@ker nanoformulation.

DOSE	I	II	III
PTX/tibia	97.5 μg	73.2 μg	48.8 μg
PTX/mouse	195 μg	146.4 μg	97.6 μg

## Data Availability

Not applicable.

## Supplementary Materials

The following supporting information can be downloaded at: https://www.mdpi.com/article/10.3390/pharmaceutics14030677/s1, Figure S1: Transmission Electron microscopy images of PTX-Ce6@ker nanoparticles; Figure S2: Chemical characterizations of PTX-Ce6@ker nanoformulation: control base line assays; Figure S3: Preclinical Osteosarcoma mouse model; Figure S4: Nanoformulation dosage and treatment’s schedule: Spectral analysis of Ce6 fluorescence in mice tissues; Figure S5: Nanoformulation dosage and treatment’s schedule: confocal analysis of Ce6 distribution; Figure S6: Nanoformulation dosage and treatment’s schedule: FLIM analysis of Ce6; Table S1: Transmission Electron Microscopy analysis; Table S2: Chemical characterizations of PTX-Ce6@ker nanoformulation: PTX release kinetics; Table S3: Chemical characterizations of PTX-Ce6@ker nanoformulation: PTX release kinetics; Table S4: Chemical characterizations of PTX-Ce6@ker nanoformulation: PTX release kinetics; Table S5: Summary of the Chlorin e6 lifetimes in all conditions tested.
