# Supplementary material for: Two Beats One: Osteosarcoma Therapy with Light-Activated and Chemo-Releasing Keratin Nanoformulation in a Preclinical Mouse Model
Source: Pharmaceutics. 2022 Mar 19;14(3):677. doi: 10.3390/pharmaceutics14030677 (PMC8950553; doi:10.3390/pharmaceutics14030677)
Supplement: Supplementary file 1 [file pharmaceutics-14-00677-s001.zip › pharmaceutics-1624343-supplementary.pdf]

pharmaceutics

Supplementary Materials: Two Beats One: Osteosarcoma Therapy with Light-Activated and Chemo-Releasing Keratin Nanoformulation in a Preclinical Mouse Model

Elisa Martella, Barbara Dozza, Claudia Ferroni, Clement Osuru Obeyok, Andrea Guerrini, Daniele Tedesco, Ilse Manet, Giovanna Sotgiu, Marta Columbaro, Marco Ballestri, Lucia Martini, Milena Fini, Enrico Lucarelli, Greta Varchi and Serena Duchi

Supplementary figures

Figure S1. Transmission Electron microscopy images of PTX-Ce6@ker nanoparticles. ____ *2*
Figure S2. Chemical characterizations of PTX-Ce6@ker nanoformulation: control base line assays ____ *2*
Figure S3. Preclinical Osteosarcoma mouse model set up. ____ *3*
Figure S4. Nanoformulation dosage and treatment's schedule: Spectral analysis of Ce6 fluorescence in mice tissues *4*
Figure S5. Nanoformulation dosage and treatment's schedule: confocal analysis of Ce6 distribution ____ *5*
Figure S6. Nanoformulation dosage and treatment's schedule: FLIM analysis of Ce6 ____ *6*

Supplementary tables

Table S1. Transmission Electron Microscopy analysis ____ *6*
Table S2. Chemical characterizations of PTX-Ce6@ker nanoformulation: PTX release kinetics ____ *6*
Table S3. Chemical characterizations of PTX-Ce6@ker nanoformulation: PTX release kinetics ____ *7*
Table S4. Chemical characterizations of PTX-Ce6@ker nanoformulation: PTX release kinetics ____ *7*
Table S5. Summary of the Chlorin e6 lifetimes in all conditions tested. ____ *8*

Supplementary figures

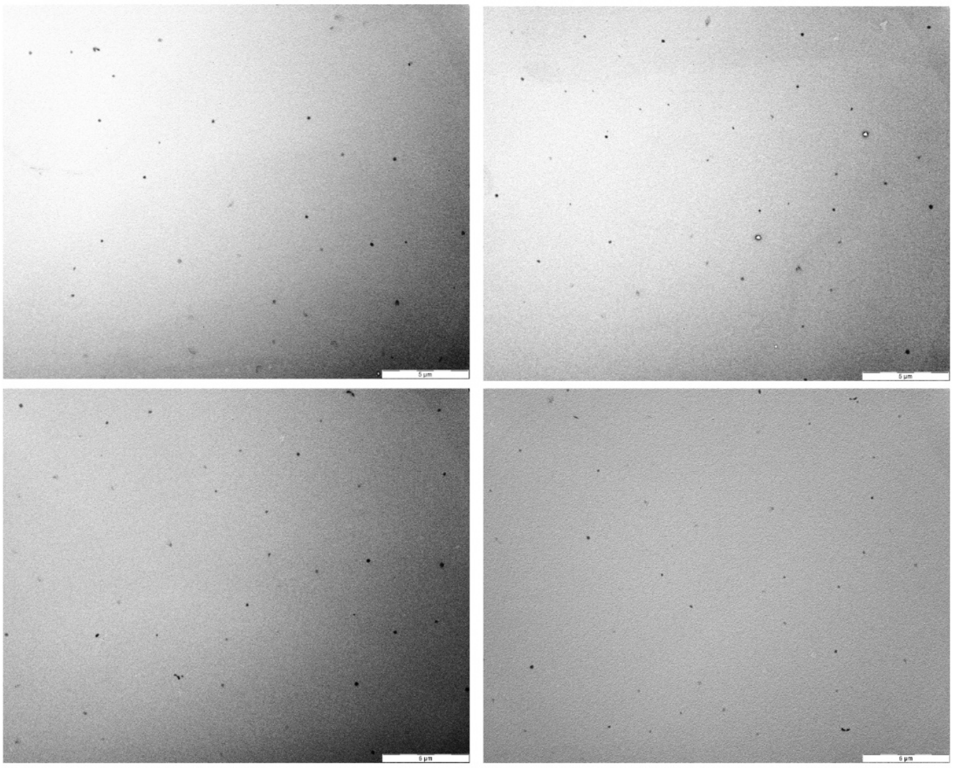

Figure S1. Transmission Electron microscopy images of PTX-Ce6@ker nanoparticles.
Four different representative Transmission electron microscopy (TEM) micrographs of PTX-Ce6@ker nanoparticles performed at a final concentration of 0.1 mg/mL; scale bar: 5µm.

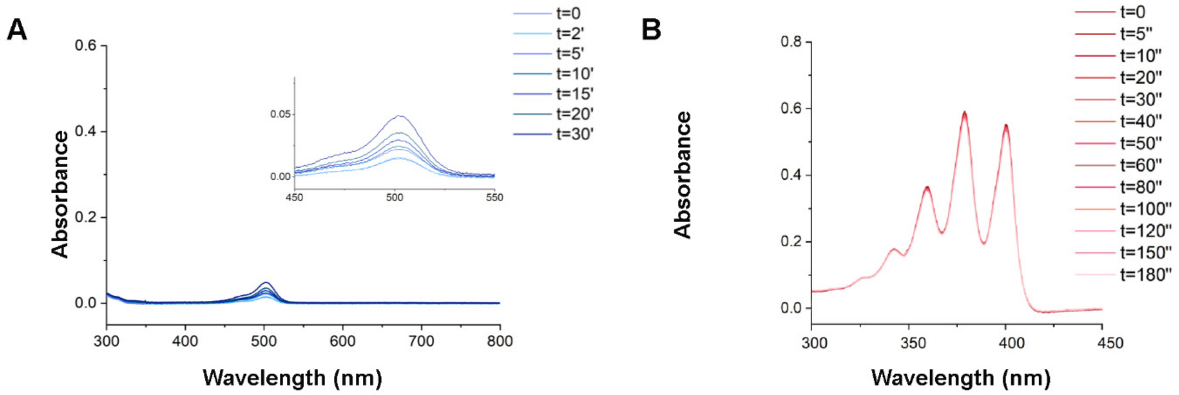

Figure S2. Chemical characterizations of PTX-Ce6@ker nanoformulation: control base line assays.
A-B The graphs show the base line profile of the dichlorofluorescein (DCF) (blue line in **A**) and the 9,10-dimethylanthracene (DMA) (red line in **B**) probes absorbance measured within the wavelength range reported in the x axes and at different irradiation times, indicated as minutes for ROS (′ in A) and seconds (″ in B) for $^1O_2$.

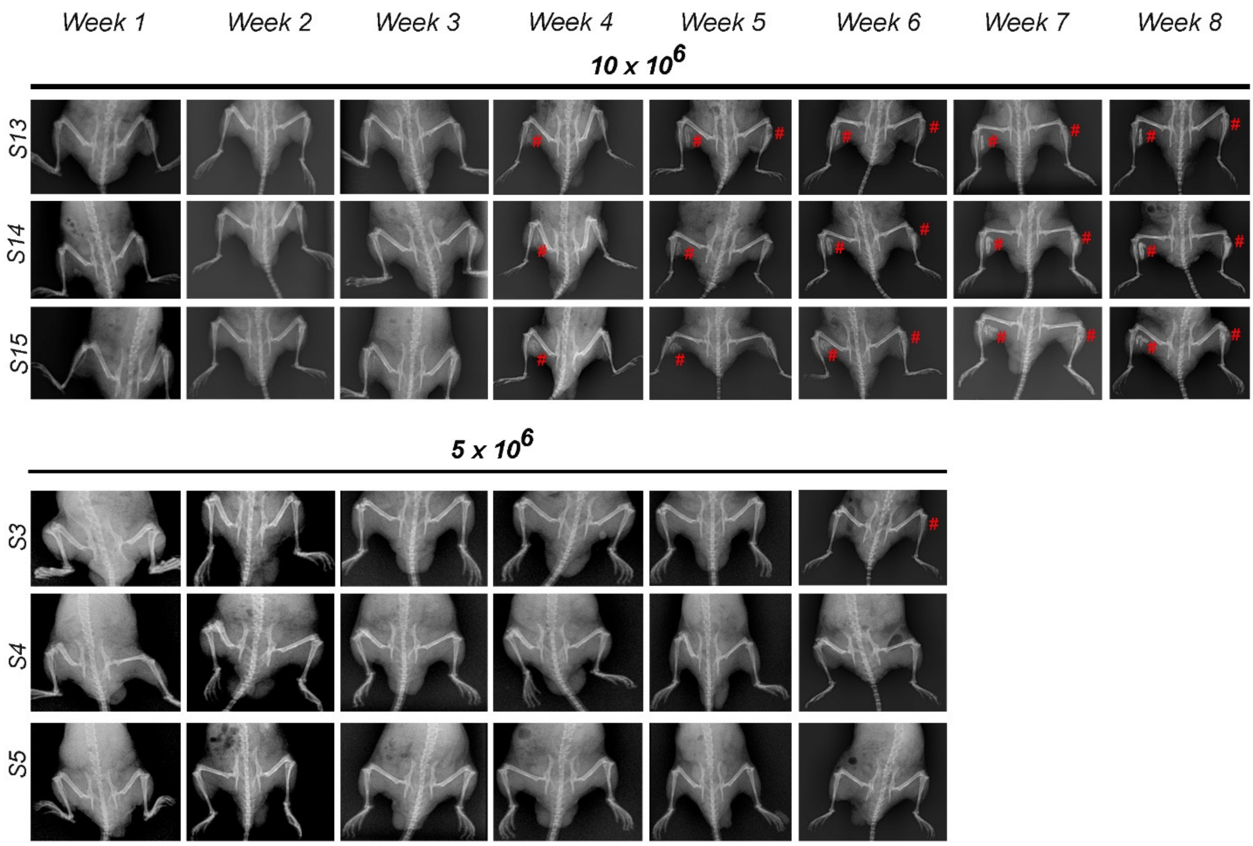

Figure S3. Preclinical Osteosarcoma mouse model set up.
Representative X-ray imaging of 6 animals (shown in the 6 parallel rows) acquired weekly up to week 8 and 6 from initial inoculation of respectively 10×10^6 and 5×10^6 Saos-2 groups, as indicated. The red hash marks point to the detectable tumor mass.

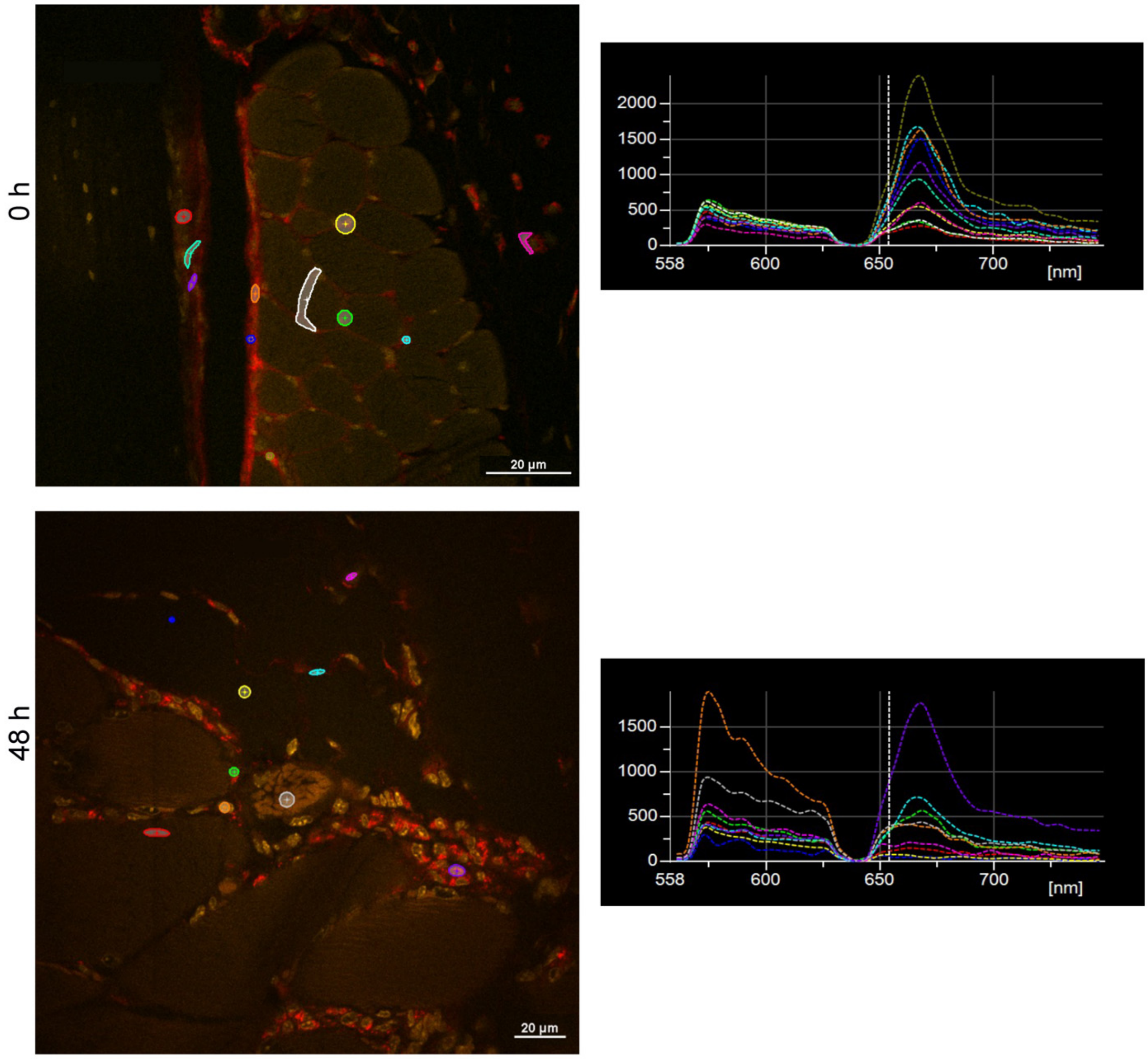

Figure S4. Nanoformulation dosage and treatment's schedule: Spectral analysis of Ce6 fluorescence in mice tissues. Representative confocal spectral images of PTX-Ce6@ker treated tissues at 0 and 48 h after nanoformulation injection. The spectral profiles of the indicated ROIs are reported in the lateral panels. Samples were excited at 405 nm. Zero-intensity at 560 nm ans 640 nm is due to dichroic mirror choice.

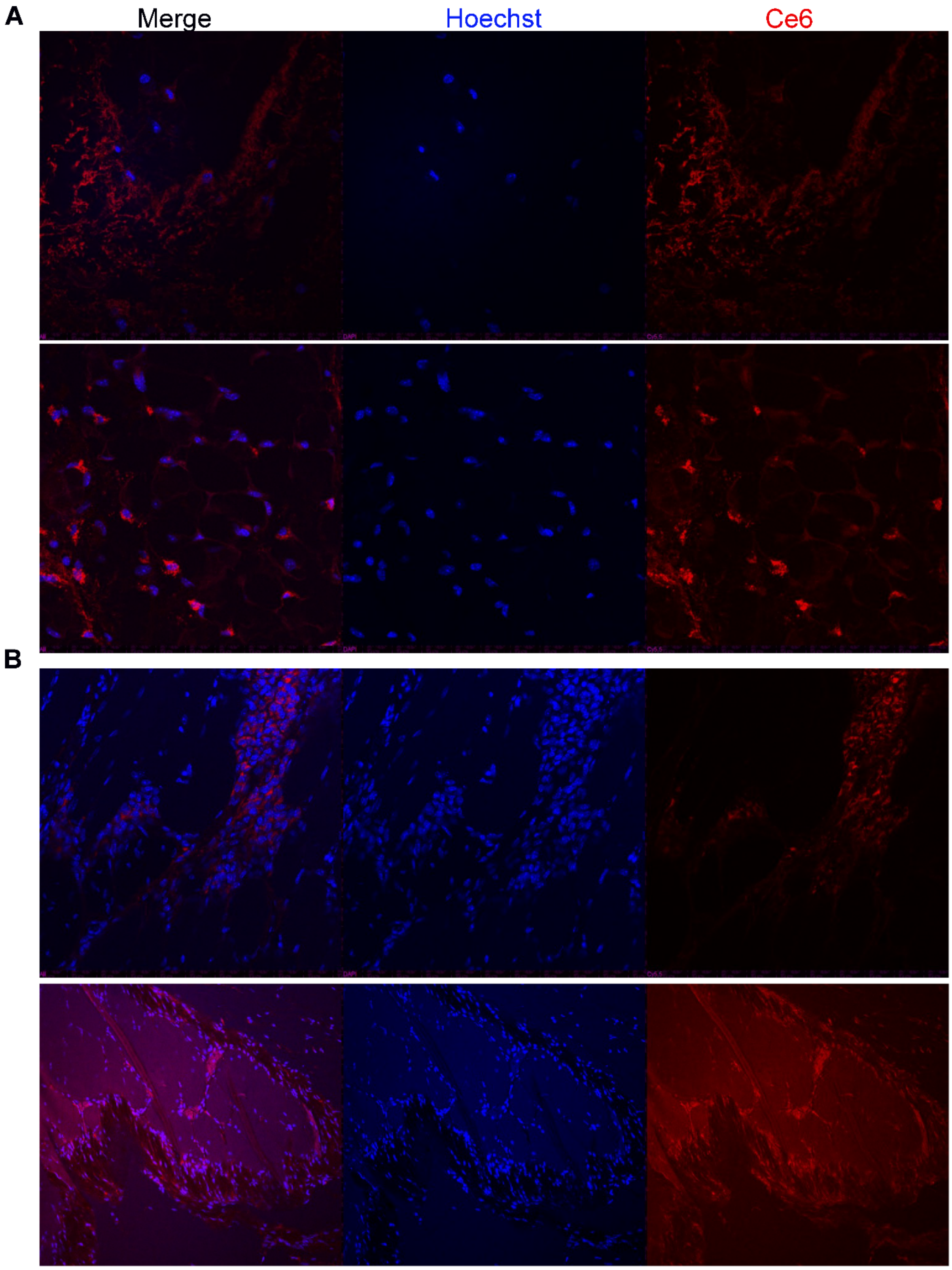

Figure S5. Nanoformulation dosage and treatment's schedule: confocal analysis of Ce6 distribution. Representative confocal fluorescence intensity images of PTX-Ce6@ker treated tissues at 0 h (A) and 48 h (B) after nanoformulation injection. Hoechst staining in shown in blue, Ce6 signal in red.

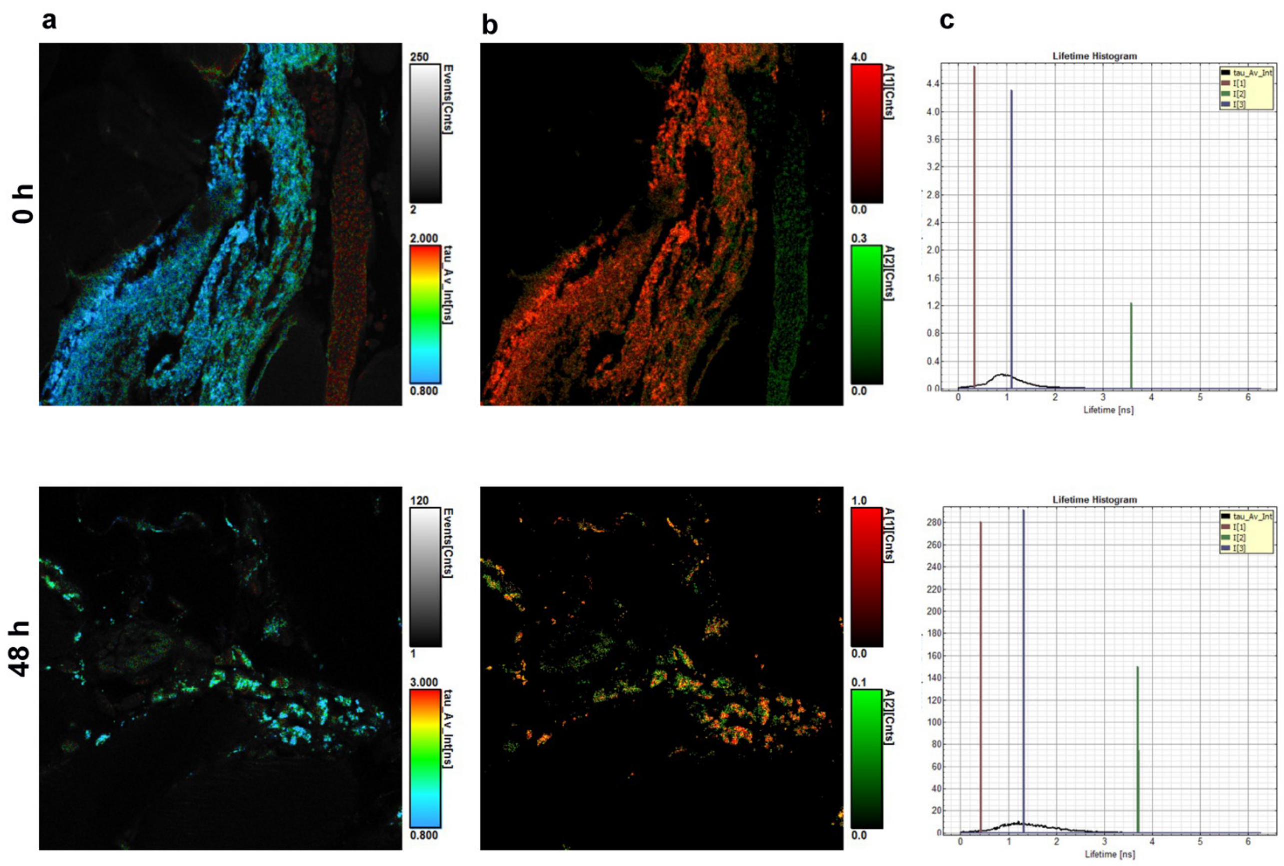

Figure S6. Nanoformulation dosage and treatment's schedule: FLIM analysis of Ce6.
Represenatitive FLIM images Ce6 signal in the samples at 0 and 48 h after nanoformulation injection. In panel **a** color scale indicates the average lifetime; panel **b** shows the pre-exponentail values of a1 and a2, the short and long lifetime respectively; panel **c** represents the lifetime histogram for the fitted area of the image.

Supplementary tables

Table S1. Transmission Electron Microscopy analysis.
Statistical details for the particle size analysis performed on PTX-Ce6@ker nanoparticles from TEM images.

**Log-normal distribution function**	$$f(x) = \frac{1}{bx\sqrt{2\pi}} e^{-\frac{(\ln x - a)^2}{2b^2}}$$
**Fitting parameters**	$a = 4.761162$<br>$b = 0.3565304$
**Mean diameter**	$\bar{x} = e^{a+\frac{b^2}{2}} = 124.55$ nm
**Standard deviation**	$\sigma_x = \sqrt{(e^{b^2} - 1)e^{2a+b^2}} = 45.86$ nm

Copyright: © 2022 by the authors. Licensee MDPI, Basel, Switzerland. This article is an open access article distributed under the terms and conditions of the Creative Commons Attribution (CC BY) license (https://creativecommons.org/licenses/by/4.0/).

Table S2. Chemical characterizations of PTX-Ce6@ker nanoformulation: PTX release kinetics.
Statistical details for the calibration curve of PTX obtained by HPLC-UV analysis.

**Calibration curve**	$f(x) = ax + b$
**Variables**	HPLC peak areas at 228 nm (µV·s) vs. PTX concentrations (µg/mL)

**Fitting parameters**	$a = 107712.5 \pm 354.5$<br>$b = -672.8 \pm 3982.0$<br>$R^2 = 0.99983$
**Limit of quantification**	$\mathrm{LOQ} = 10\frac{\sigma_b}{a} = 0.370\ \mu\mathrm{g/mL}$

Table S3. Chemical characterizations of PTX-Ce6@ker nanoformulation: PTX release kinetics.
PTX release data determined by HPLC-UV analysis during the dialysis of PTX-Ce6@ker nanoparticles against [PBS, pH 7.4]/ethanol 75:25, v/v.

Dialysis time (h)	Released PTX (w/w)
**0.5**	10.29 ± 0.07%
**1.0**	14.49 ± 0.14%
**1.5**	18.54 ± 0.03%
**2.0**	21.95 ± 0.11%
**2.5**	26.09 ± 0.18%
**3.0**	29.46 ± 0.14%
**4.5**	38.77 ± 0.04%
**6.0**	46.83 ± 0.07%
**7.5**	53.41 ± 0.06%
**9.0**	58.98 ± 0.17%
**21.5**	80.57 ± 0.30%
**24.0**	81.24 ± 0.08%
**26.5**	82.32 ± 0.27%
**29.0**	82.96 ± 0.19%

Table S4. Chemical characterizations of PTX-Ce6@ker nanoformulation: PTX release kinetics.
Statistical details for the PTX release kinetics analysis on PTX-Ce6@ker nanoparticles

**Cumulative Weibull distribution function**	$\boldsymbol{f_{\mathrm{PTX}} = 1 - e^{-\alpha t^{\beta}}}$
**Fitting parameters**	$\alpha = 0.1621 \pm 0.0067$<br>$\beta = 0.7398 \pm 0.0178$<br>$R^2 = 0.99628$
**Release half-time**	$t_{½} = \left(\frac{\ln 2}{\alpha}\right)^{\frac{1}{\beta}} = 7.128\ \mathrm{h}$
**Korsmeyer-Peppas model**<br>**Time range**	$f_{\mathrm{PTX}} = k_P t^{n_P}$<br>$0.5\ \mathrm{h} \le t \le 9.0\ \mathrm{h}$ ($f_{PTX} < 60\%$, w/w)
**Fitting parameters**	$k_P = 0.1461 \pm 0.0025$<br>$n_P = 0.6406 \pm 0.0097$<br>$R^2 = 0.99875$

Table S5. Summary of the Chlorin e6 lifetimes in all conditions tested.

	τ_1 / ns	τ_2 / ns	τ_3 / ns	τ_{av} / ns
**Solution Ce6 in PBS**	3.8			
**Solution PTX-Ce6@ker**	1.8	4.3		4.1
**Tissue PTX-Ce6@ker**				
**0 h cytoplasm**	0.3	1.1	3.5	0.9
**0 h intercellular**	0.3	1.1	3.6	0.8
**48 h cytoplasm**	0.4	1.3	3.7	1.1
